# Combined CT and serum CA19-9 for stratifying risk for progression in patients with locally advanced pancreatic cancer receiving intraoperative radiotherapy

**DOI:** 10.3389/fonc.2023.1155555

**Published:** 2023-04-14

**Authors:** Wei Cai, Yongjian Zhu, Ze Teng, Dengfeng Li, Qinfu Feng, Zhichao Jiang, Rong Cong, Zhaowei Chen, Siyun Liu, Xinming Zhao, Xiaohong Ma

**Affiliations:** ^1^ Department of Diagnostic Radiology, National Cancer Center/National Clinical Research Center for Cancer/Cancer Hospital, Chinese Academy of Medical Sciences and Peking Union Medical College, Beijing, China; ^2^ Department of Radiation Oncology, National Cancer Center/National Clinical Research Center for Cancer/Cancer Hospital, Chinese Academy of Medical Sciences and Peking Union Medical College, Beijing, China; ^3^ Department of Medical Oncology, National Cancer Center/National Clinical Research Center for Cancer/Cancer Hospital, Chinese Academy of Medical Sciences and Peking Union Medical College, Beijing, China; ^4^ Magnetic Resonance Imaging Research, General Electric Healthcare (China), Beijing, China

**Keywords:** locally advanced pancreatic cancer, intraoperative radiotherapy, prognosis, progression, computed tomography, carbohydrate antigen 19-9

## Abstract

**Background and purpose:**

The aim of this study was to evaluate the significance of baseline computed tomography (CT) imaging features and carbohydrate antigen 19-9 (CA19-9) in predicting prognosis of locally advanced pancreatic cancer (LAPC) receiving intraoperative radiotherapy (IORT) and to establish a progression risk nomogram that helps to identify the potential beneficiary of IORT.

**Methods:**

A total of 88 LAPC patients with IORT as their initial treatment were enrolled retrospectively. Clinical data and CT imaging features were analyzed. Cox regression analyses were performed to identify the independent risk factors for progression-free survival (PFS) and to establish a nomogram. A risk-score was calculated by the coefficients of the regression model to stratify the risk of progression.

**Results:**

Multivariate analyses revealed that relative enhanced value in portal-venous phase (REV-PVP), peripancreatic fat infiltration, necrosis, and CA19-9 were significantly associated with PFS (all *p* < 0.05). The nomogram was constructed according to the above variables and showed a good performance in predicting the risk of progression with a concordance index (C-index) of 0.779. Our nomogram stratified patients with LAPC into low- and high-risk groups with distinct differences in progression after IORT (*p* < 0.001).

**Conclusion:**

The integrated nomogram would help clinicians to identify appropriate patients who might benefit from IORT before treatment and to adapt an individualized treatment strategy.

## Introduction

Pancreatic ductal adenocarcinoma (PDAC) is a highly aggressive malignant tumor that results in many deaths as new cases (1, 2). Approximately 30-35% of patients were diagnosed with locally advanced pancreatic cancer (LAPC) based on the relationship between the primary tumor and the adjacent blood vessels (2, 3). Despite improvements in therapeutic approaches in recent years, the 5-year survival rate of PDAC is only approximately 10% (3).

With the improvement and optimization of chemotherapy regimens, the systemic conditions and distal metastases of LAPC could be controlled effectively. However, approximately 30% of LAPC patients died from local progression during the period of systemic therapy (4). Therefore, improved local control might provide more benefits to LAPC patients. Radiotherapy is proven to be an effective local treatment that can improve the local control rate and delay local progression, according to previous clinical studies (2, 5). Intraoperative radiotherapy (IORT), a more targeted form of radiotherapy, improves this effect by delivering high doses of irradiation to the target area, resulting in a higher rate of local control compared with conventional external radiation therapy. IORT has been proven to reduce complications, relieve pain, improve quality of life, and possibly prolong survival in LAPC (6–9). Experts’ Consensus on IORT for PDAC established a standard protocol for IORT and identified LAPC as an indication for IORT (10). As well, the European Society for Radiotherapy and Oncology-Advisory Committee on Radiation Oncology Practice (ESTRO-ACROP) recommends IORT for unresectable locally progressive pancreatic cancer (11).

Nevertheless, the prognosis of LAPC patients after IORT varied significantly due to the heterogeneity and complexity of pancreatic cancer (12). Instead of benefiting, patients who are insensitive to IORT might progress rapidly after surgery and suffer a series of complications, toxicities, and financial losses (10). Therefore, it is of great clinical significance to accurately identify the appropriate individuals who could benefit from IORT before treatment.

Contrast-enhanced computed tomography (CECT), the most commonly used imaging technique for the depiction, staging, and assessment of the resectability of PDAC (2, 13, 14), could provide tumor biological and pathological information, including semantic features such as necrosis and peripancreatic tumor infiltration (15) and quantitative parameters such as tumor size and attenuation values (16, 17). Furthermore, CT imaging features have been reported as imaging markers of treatment efficiency and prognosis (15, 16). Serum tumor markers, such as carbohydrate antigen 19-9 (CA19-9), carcinoembryonic antigen (CEA), have been reported to be associated prognosis in PDAC (18, 19). Cai et al. found that the CT attenuation values of PDAC could help stratify the aggressiveness and prognosis (16). In addition, Marchegiani et al. reported that CT attenuation value changes could help identify the possibility of R0 resection after neoadjuvant therapy in locally advanced and borderline resectable pancreatic cancer (20). CA19-9 is a well-known serum biomarker for PDAC, and its level is correlated with tumor burden (21). A high preoperative CA19-9 level has been reported to be associated with severe tumor burden, low differentiation, and poor prognosis (18, 21). To date, there are only a few studies focused on the imaging evaluation of IORT response and prognosis of pancreatic cancer (22–24). To our knowledge, the value of CT combined with serum CA19-9 in predicting the prognosis of LAPC patients receiving IORT has not been fully clarified.

Therefore, the purpose of this study was to evaluate the significance of baseline CT imaging features and serum CA19-9 in predicting the risk of progression of LAPC receiving IORT and to establish an objective, simple, and clinically practical progression risk nomogram by integrating CT imaging features and CA19-9. This would assist clinicians to identify appropriate patients who would benefit from IORT before treatment and to adapt an individualized treatment strategy.

## Materials and methods

### Patients

**Figure 1:**
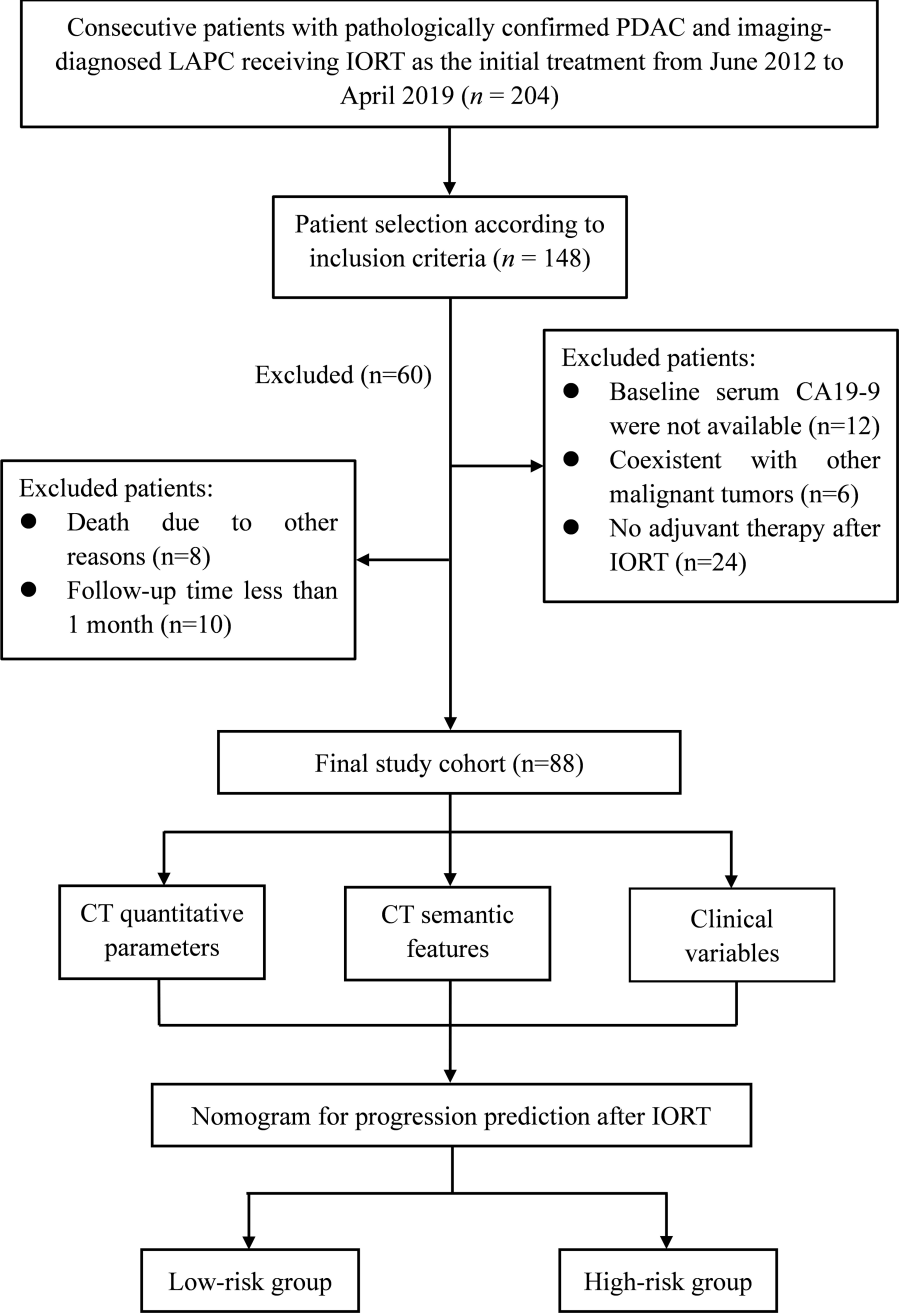
Flow chart of the patient enrollment process and illustration of this study. LAPC, locally advanced pancreatic cancer; IORT, intraoperative radiotherapy.

The Institutional Review Board approved (IBR) this retrospective study, waiving the requirement for informed consent because of the retrospective study design (IBR number: 21/412-2608). Between June 2012 and April 2019, we retrospectively searched the medical record database in our institutional to collect the consecutive patients with pathologically confirmed PDAC based on imaging, with IORT as the initial treatment modality (n = 204). The definition of LAPC was in accordance with the NCCN guideline (2). The following inclusion criteria resulted in 148 participants: (a) underwent three-phase CECT examinations dedicated to the pancreas within 2 weeks before IORT; (b) regular follow-up after IORT. Among these patients, 60 were excluded for the following reasons: (a) no adjuvant therapy (chemotherapy or chemoradiotherapy) after IORT (n=24); (b) baseline serum CA19-9 was not available (n=12); (c) coexistence with other malignant tumors (n=6); (d) death due to other reasons (n=8); (e) follow-up time less than 1 month (n=10). The patient recruitment process and study design were depicted in **Figure 1**.

### Clinical data collection

Clinical data were routinely collected, including age, sex, treatment type, jaundice, American Joint Committee on Cancer (AJCC^8th^) TNM stage, CA19-9, CEA, carbohydrate antigen 242 (CA242), total and direct bilirubin, albumin (ALB), D-dimer, fibrinogen, glucose, and transferrin. Since serum CA19-9 level might be affected by jaundice (25). Endoscopic nasobiliary drainage (ENBD) was performed for biliary drainage on jaundiced patients. The cut-off value of laboratory tests is all based on the normal range at our hospital.

### IORT and adjuvant therapy

**Figure 2:**
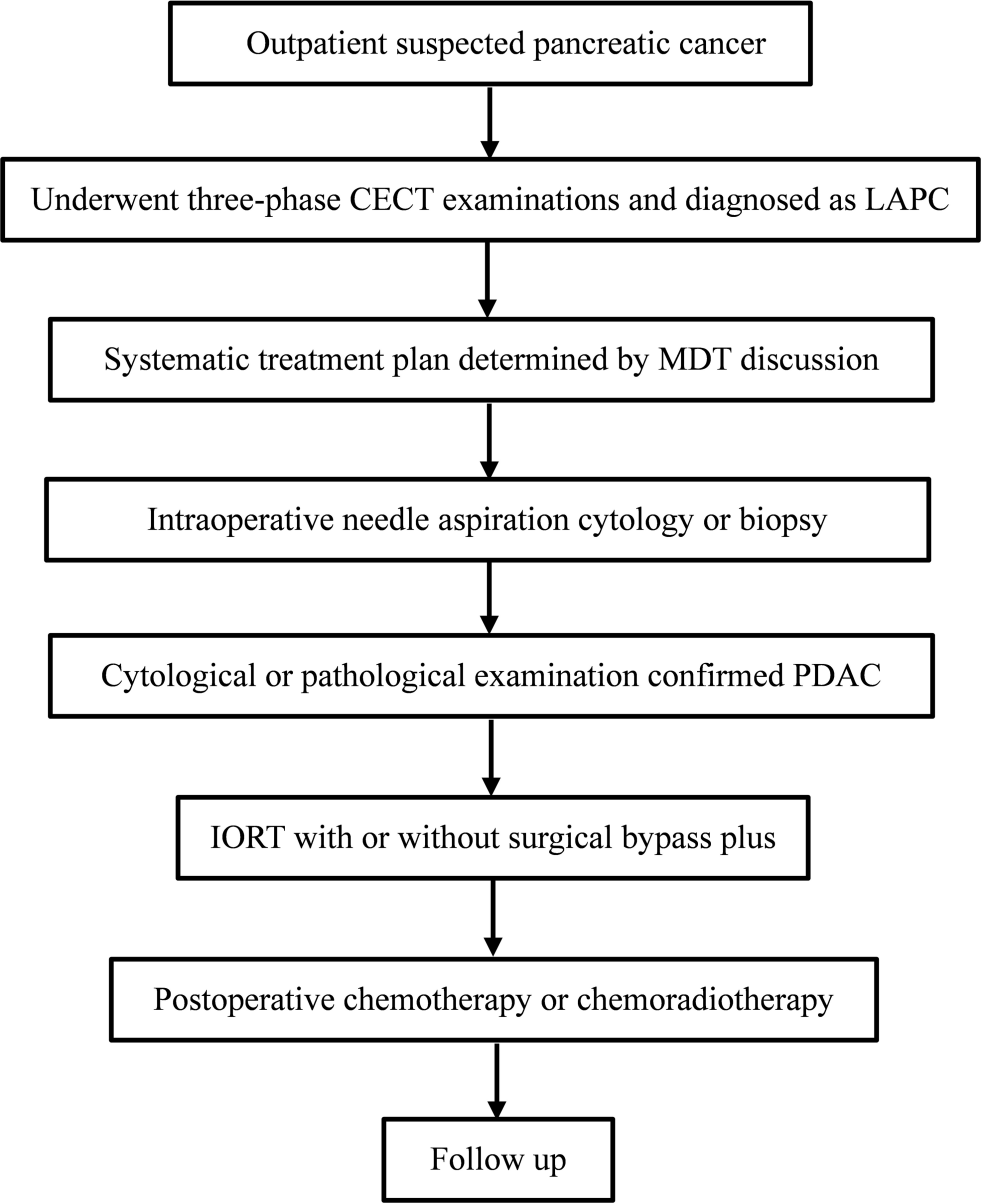
The illustration of IORT procedure in LAPC. IORT, intraoperative radiotherapy; LAPC, locally advanced pancreatic cancer; CECT, contrast-enhanced CT; MDT, multi-disciplinary team; PDAC, pancreatic ductal adenocarcinoma.

The IORT procedure and sequential adjuvant therapy strategy were determined in accordance with a standardized protocol reported by experts’ consensus (10) and established by the abdominal radiation oncology team at our institution. The illustration of the IORT procedure in LAPC was shown in **Figure 2**. Surgical bypass, including biliary bypass and gastrojejunostomy, might be performed before IORT depending on the tumor location and clinical symptoms. Details of the treatment plan can be seen in **Supplementary Appendix S1** and **Supplementary Table S1**.

### CT protocol

Multiphase CECT examinations consisting of non-enhanced (N), arterial phase (AP), pancreatic parenchymal phase (PPP), and portal venous phase (PVP), were performed on all patients. Impromide (Ultravist, Schering, Berlin, Germany) was administered to each patient at a rate of 4 mL/sec, with a weight-dependent dose of 1.5 mL/kg. AP, PPP, and PVP were defined as 25-30 sec, 40-50 sec, and 65-70 sec, respectively, after contrast injection. Images were routinely generated at 5.0 mm thickness in the axial plane in all phases for radiographic evaluation. Given the time span of the study, the CT examinations were carried out on different instruments. Details of the CT scanner parameters are showed in **Supplementary Table S2**.

### Imaging analysis

Two abdominal radiologists (with 10 and 6 years of experience, respectively) who were aware of the diagnosis of PDAC but blinded to the clinical details, independently reviewed the CT images. The following CT semantic features were evaluated: tumor attenuation in four phases, location, necrosis, rim-enhancement, peripancreatic fat infiltration, pancreatic duct dilatation, atrophic upstream pancreatic parenchyma, suspicious lymph nodes, according to the PDAC radiology reporting template proposed by the Society of Abdominal Radiology and previous studies (26–29). The definition of these features was summarized in **Supplementary Table S3**.

**Figure 3:**
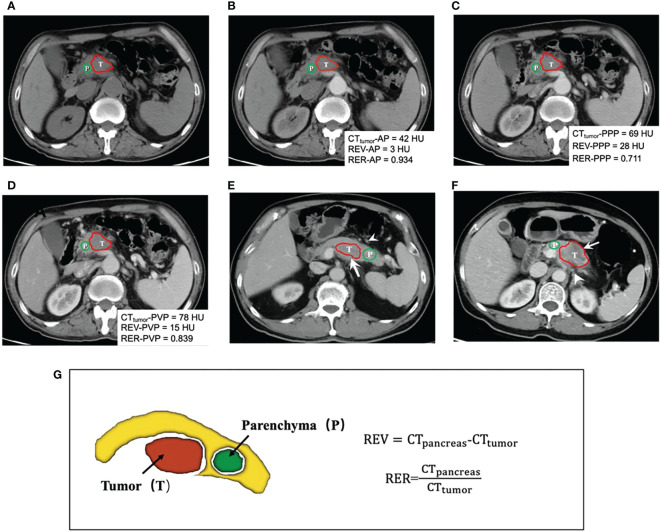
**(A-D)** shows a 58-year-old man with 4.5cm LAPC at uncinate of the pancreas in non-enhanced (N), arterial phase (AP), pancreatic parenchymal phase (PPP) and portal venous phase (PVP) before IORT. Quantification of CT attenuation values, relative enhanced value (REV) and relative enhanced ratio (RER) were calculated and displayed in the images. This patient was classified in the low-risk group. Finally, PFS time of this patients was 10.8 months after IORT and the final progression pattern was liver metastasis. CT images **(E)** in PVP in a 61-year-old man with a 4.2cm lesion appearing hypo-attenuation at the body of pancreas (arrow). The patient was at low risk of progression, without necrosis and peripancreatic fat infiltration (arrowhead). Subsequently, he was found local progression after 9.6 months of IORT. CT images **(F)** in PVP in a 64-year-old woman with 5.3cm LAPC at the body of pancreas. In this case, peripancreatic fat infiltration (arrowhead) and necrosis (arrow) could be observed. This patient was finally assessed as a high-risk group for progression. After 2.7 months of IORT, the patient was found to have peritoneal metastasis. Schematic diagram **(G)** demonstrated delineation of tumor (red), the surrounding normal pancreatic parenchyma (yellow), and delineation of normal pancreatic parenchyma (green). The formulas of CT quantitative parameters are displayed. Red and green lines show the delineation of tumor lesion and the normal peripancreatic parenchyma, respectively. T indicated the tumor and P represented surrounding normal peripancreatic parenchyma. REV, relative enhanced value; RER, relative enhanced ratio.

Quantitative CT parameters, including long and short diameters in PVP, relative enhanced value (REV), and relative enhanced ratio (RER) in the three phases, were measured and calculated as previous study reported (16). The specific definitions are detailed in **Figure 3** and **Supplementary Appendix S2**.

### Follow-up

After IORT, all patients were closely followed up through outpatient clinic visit. Physical examinations and laboratory tests were performed monthly. Imaging examinations, including CT or MRI, were performed every 3 months. Progression was defined as tumor local progression or distal metastasis confirmed by pathology or imaging, and any disease-related death. Local progression was defined ≥ 20% increase in tumor size of tumor lesions or the appearance of new lesions according to RECIST v1.1 criteria (30). The progression-free survival (PFS) time was defined as the interval between IORT and the first day of confirmed progression or the last follow-up without progression. All patients were observed for progression until the final follow-up date of June 30, 2019.

### Statistical analysis

Continuous variables were compared using the independent *t* test or Mann-Whitney *U* test, and categorical variables were analyzed using *χ* ^2^ or Fisher exact test as appropriate. Consistency between readers was evaluated using Cohen kappa statistics for CT semantic features and the intraclass correlation coefficient (ICC) for quantitative parameters.

Univariate Cox proportional hazards analysis was performed to evaluate the association between PFS and variables. Variables with *p* < 0.10 in univariate analysis, in which continuous variables were converted to a binary classification for clinical convenience, were entered into the multivariate analysis by using a forward stepwise method to identify significantly independent risk factors for PFS. A simple nomogram was established based on the multivariate Cox regression analysis to predict the individual probability of PFS. The Harrell’s concordance index (C-index) and calibration curve were used to evaluate the nomogram’s performance. Decision curve analysis was used to assess the clinical usefulness of the nomogram. We also evaluated the performance of this nomogram to predict the probability of the PFS, quantified by sensitivity, specificity, positive predictive value (PPV), and negative predictive value (NPV).

A risk-score was generated *via* the summing of the independent prognostic factors weighted by their respective coefficients. The patients were classified into the high-risk and low-risk groups according to the risk-score. The outcome-based optimal cut-off value for REV and RER were determined using the maximally selected rank statistics (Maxstat package) in R statistical software. Survival curve analysis was generated by the Kaplan-Meier method, and the log-rank test was used to compare between different risk groups. All statistical analyses were conducted using R (version 3.6.3, R Foundation for Statistical Computing, Vienna, Austria). *p* < 0.05 was considered statistical significance.

## Results

### Patient and follow-up

A total of 88 patients (mean age, 59 years ± 9 [SD]) including 50 men and 38 women were included. The baseline demographics and clinical characteristics are summarized in **Table 1**. No patients received radical surgery after IORT plus adjuvant therapy based on multidisciplinary discussion due to the poor performance status of the patients.

**Table 1:** Baseline clinical characteristics and univariate analysis for PFS.

Characteristic	N (%) or Mean ± SD^*^	Univariate analysis	
	(Total, n = 88)	HR (95% CI)	*p* Value^**^
Age (years)	59 ± 9	0.996 (0.973–1.018)	0.703
Sex			
Male	50 (56.8)	Reference	
Female	38 (43.2)	1.045 (0.632–1.484)	0.839
IORT Radiation dose	14.5 ± 0.73	0.78 (0.57-1.08)	0.201
Adjuvant Therapy			
Chemotherapy	56 (63.6)	Reference	
Chemoradiotherapy	32 (36.4)	0.981 (0.632–1.524)	0.932
AJCC 8^th^ T stage			
T1-2	48 (54.5)	Reference	
T3-4	40 (45.5)	1.131 (0.967–1.532)	0.123
AJCC 8^th^ N stage			
N0	54 (61.4)	Reference	0.725
N1	8 (9.1)	0.958 (0.414–1.905)	0.837
N2	26 (29.5)	1.259 (0.831–1.927)	0.453
Jaundice			
Absent	52 (59.1)	Reference	
Present	36 (41.9)	1.322 (0.833–2.101)	0.236
BMI (kg/m^2^)	25.7 (22.0–30.1)	1.038 (0.986–1.094)	0.153
CA 19-9 (U/ml)			
Normal (< 37 U/ml)	16 (18.2)	Reference	**<0.001**
Abnormal (≥ 37 U/ml)	72 (81.8)	3.073 (1.711-5.521)	
CEA (ng/ml)			
Normal (< 5 ng/ml)	57 (64.7)	Reference	0.322
Abnormal (≥ 5 ng/ml)	31 (35.3)	0.794 (0.504–1.253)	
CA 242 (U/ml)			
Normal (< 20 U/ml)	40 (45.5)	Reference	0.401
Abnormal (≥ 20 U/ml)	48 (54.5)	1.200 (0.784-1.837)	
Total bilirubin (μmol/L)			
Normal (< 26 μmol/L)	55 (62.5)	Reference	0.709
Abnormal (≥ 26 μmol/L)	33 (37.5)	1.088(0.698-1.698)	
Direct bilirubin(μmol/L)			
Normal (< 4 μmol/L)	17 (19.3)	Reference	0.456
Abormal (> 4 μmol/L)	71 (80.7)	1.224(0.719-2.084)	
D-dimer (mg/L)			
Normal (< 0.55 mg/L)	55 (62.5)	Reference	0.900
Abnormal (≥ 0.55 mg/L)	33 (37.5)	0.971(0.616-1.532)	
Fibrinogen (g/L)			
Normal (< 4.35 g/L)	72 (81.2)	Reference	0.911
Abnormal (≥ 4.35 g/L)	16 (18.2)	1.033(0.589-1.810)	
Glucose (mmol/L)			
Normal (< 6.1 mmol/L)	48 (54.5)	Reference	0.393
Abnormal (≥ 6.1 mmol/L)	40 (45.5)	0.831(0.544-1.270)	
Transferrin (mg/dl)			
Normal (< 400 mg/dl)	69 (78.4)	Reference	0.741
Abnormal (≥ 400 mg/dl)	19 (21.6)	1.090(0.653-1.820)	
Albumin (g/L)			
Normal (≥ 40 g/L)	63 (71.6)	Reference	**<0.001**
Abnormal (< 40 g/L)	25 (28.4)	3.418(2.019-5.785)	

Statistically significant results are marked in bold.

PFS, progression-free survival; CI, confidence interval; HR, hazard ratio; IORT, Intraoperative radiotherapy; AJCC, American Joint Committee on Cancer; BMI, body mass index; CA19-9, carbohydrate antigen 19-9; CEA, carcinoembryonic antigen; CA242, cancer antigen 242.

^*^Data are reported as mean ± standard deviation or median with interquartile range in parentheses for continuous variables, and number (%) of patients for category variables, as appropriate.

^**^p values were calculated via univariate cox proportional hazard analysis.

The median follow-up time was 5.14 months (range, 1.53–37.86 months). During follow-up, all patients developed disease progression after IORT. Distant metastases occurred in most patients (52/88, 59.1%), followed by local progression (19/88, 21.6%), and both in the remaining individuals (17/88, 19.3%). Detailed progression pattern was shown in **Supplementary Table S4**. In the whole cohort, the median PFS time was 4.30 months (95% confidence interval [CI]: 2.89–5.71 months), while the PFS rates at 3 months, 6 months, and 1 year were 68.2%, 38.6%, and 15.9%, respectively.

### Quantitative CT parameters and semantic feature

The quantitative CT parameters and semantic features are summarized in **Table 2**. The relationship between tumor and peripheral vascular was supplied in **Supplementary Table S5**. The κ values for the semantic features were 0.65–1.00 and the ICCs for the quantitative CT parameters were 0.79–0.87, both of which indicated moderate-to-excellent inter-reader agreement (**Supplementary Table S6**).

**Table 2:** Imaging features and univariate analysis for PFS.

Features	N (%) or Mean ± SD^*^	Univariate analysis	
	(Total, n = 88)	HR (95% CI)	*p* Value^**^
Quantitative parameters			
Long-axis			
2 cm–4 cm	53 (60.2)	Reference	
> 4 cm	35 (39.8)	1.497 (0.972–2.304)	0.108
Short-axis			0.307
≤ 2 cm	19 (21.6)	Reference	
2 cm–4 cm	61 (69.3)	1.459 (0.864–2.466)	0.158
> 4 cm	8 (9.1)	1.695 (0.728–3.943)	0.221
CT_tumor_ -AP (HU)	45.0 (41.25–51.0)	0.988 (0.959–1.018)	0.422
CT_tumor_ -PPP (HU)	61.0 (56.0–67.75)	0.981 (0.956–1.006)	0.129
CT_tumor_ -PVP (HU)	70.0 (64.0–79.0)	0.988 (0.972–1.004)	0.131
REV-AP (HU)	19.5 (12.0–33.0)	0.998 (0.989–1.007)	0.724
REV-PPP (HU)	36.1 ± 16.9	1.009 (0.996–1.022)	0.157
REV-PVP (HU)	23.0 (15.25–35.0)	1.054 (1.035–1.073)	**<0.001**
RER-AP	0.68 (0.57–0.79)	1.256 (0.416–3.792)	0.686
RER-PPP	0.61 ± 0.15	0.331 (0.078–1.408)	0.135
RER-PVP	0.63 (0.52–0.74)	0.169 (0.033–0.865)	**0.033**
Semantic features			
Tumor Location			
Head/uncinate	63 (71.6)	Reference	
Body/tail	25 (28.4)	0.821 (0.512–1.316)	0.412
N			
Iso-attenuating	68 (77.3)	Reference	
Hypo-attenuating	20 (22.7)	1.275 (0.768–2.118)	0.348
AP			
Iso-attenuating	26 (29.5)	Reference	
Hypo-attenuating	62 (70.5)	1.162 (0.731–1.847)	0.525
PPP			
Iso-attenuating	15 (17.0)	Reference	
Hypo-attenuating	73 (83.0)	1.051 (0601–1.837)	0.862
PVP			
Iso-attenuating	20 (22.7)	Reference	
Hypo-attenuating	68 (77.3)	1.356 (0.806–2.280)	0.251
Necrosis			
Absent	49 (55.7)	Reference	
Present	39 (44.3)	1.573 (1.019–2.428)	**0.041**
Rim-enhancement			
Absent	67 (76.1)	Reference	
Present	21 (23.9)	1.163 (0.710–1.904)	0.550
Peripancreatic fat infiltration			
Absent	49 (55.7)	Reference	
Present	39 (44.3)	2.672 (1.693–4.217)	**<0.001**
Suspicious lymph nodes			
Absent	54 (61.4)	Reference	
Present	34 (38.6)	1.130 (0.732–1.745)	0.580
Pancreatic duct dilatation			
Absent	49 (55.7)	Reference	
Present	39 (44.3)	1.020 (0.664–1.566)	0.928
Atrophic upstream pancreatic parenchyma			
Absent	42 (47.7)	Reference	
Present	46 (52.3)	1.149 (0.747–1.768)	0.527

Statistically significant results are marked in bold.

PFS, progression-free survival; CI, confidence interval; HR, hazard ratio; CT_tumor_, the CT attenuation value of tumor; REV, relative enhanced value; RER, relative enhanced ratio; N, non-enhanced; AP, arterial phase; PPP, pancreatic parenchymal phase; PVP, portal venous phase.

^*^Data are reported as mean ± standard deviation or median with interquartile range in parentheses for continuous variables, and number (%) of patients for categoric variables, as appropriate.

^**^p values were calculated via univariate cox proportional hazard analysis.

### Identification of variables for progression prediction in LAPC receiving IORT

Univariate Cox proportional hazards analysis found that REV-PVP, RER-PVP, necrosis, peripancreatic fat infiltration, serum CA19-9 level, and ALB might be associated with PFS (**Tables 1**, **2**).

**Figure 4:**
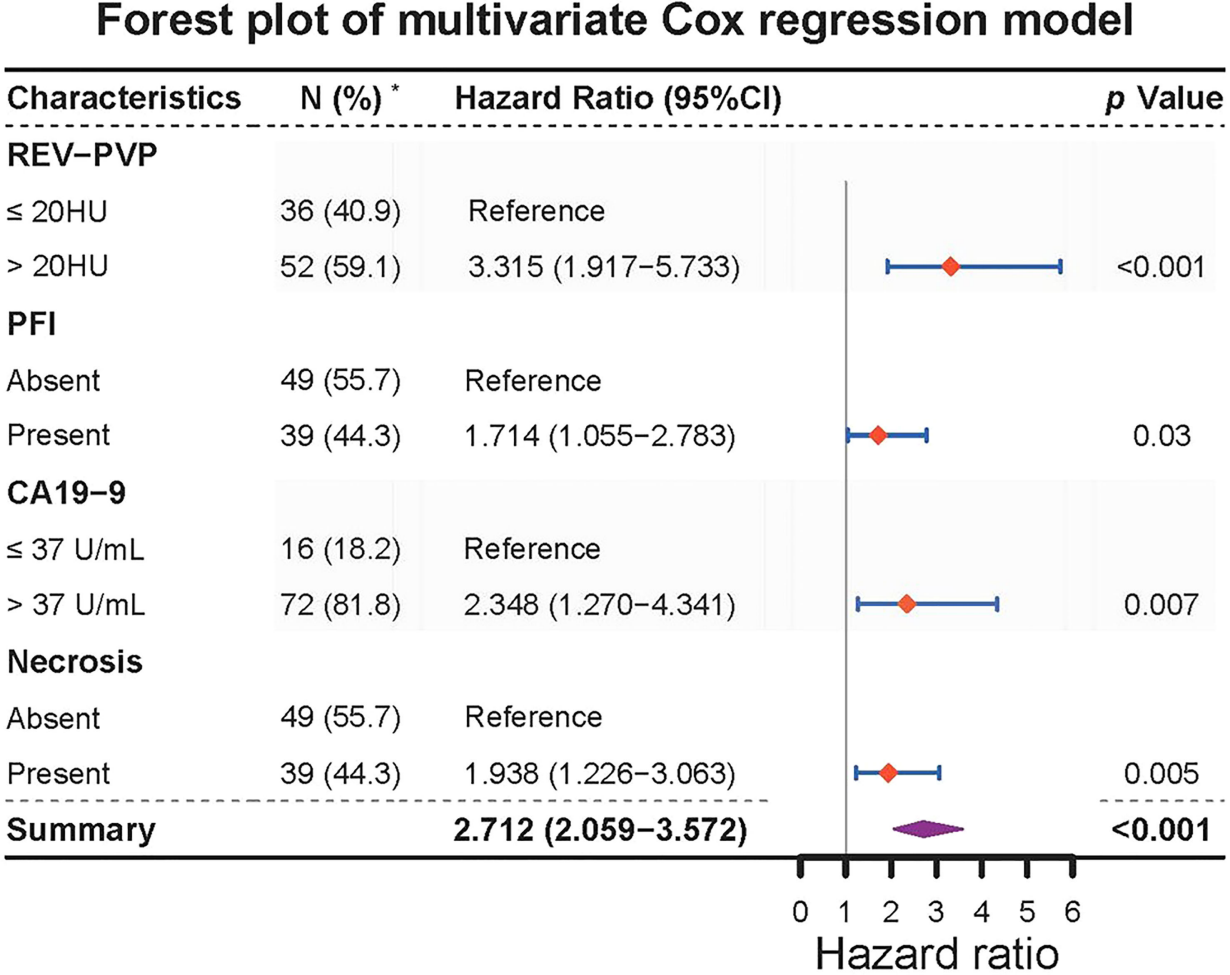
Forest plot of multivariate Cox regression model for progression-free survival in 88 LAPC patients. REV, relative enhanced value; PVP, portal venous phase. PFI, peripancreatic fat infiltration. PFS, progression-free survival; CI, confidence interval; REV, relative enhanced value; RER, relative enhanced ratio; PVP, portal venous phase; CA19-9, carbohydrate antigen 19-9.* The content in parentheses of parameter indicates the number and percentage of patients.

The optimal cut-off values of REV-PVP and RER-PVP were 20 HU and 0.716, respectively. Ultimately, REV-PVP > 20 HU (hazard ratio [HR] = 3.315, 95% CI = 1.917–5.733, *p* < 0.001), peripancreatic fat infiltration (HR = 1.714, 95% CI = 1.055–2.783, *p* = 0.009), necrosis (HR = 1.938, 95% CI = 1.226–3.063, *p* = 0.030) and abnormal serum CA19-9 level (HR = 2.348, 95% CI = 1.270–4.341, *p* = 0.007) were independent risk factors for PFS through multivariate Cox analysis (**Table 3** and **Figure 4**).

**Table 3:** Multivariate cox proportional hazard analysis for PFS of LAPC patients.

Variables	Multivariate analysis^*^	
	HR (95% CI)	*p* Value
CA19-9 (U/mL)		
Normal (≤ 37 U/mL)	Reference	0.007
Abnormal (> 37 U/mL)	2.348 (1.270–4.341)	
Albumin (g/L)		
Normal (≥35 g/L)	Reference	…
Abnormal (< 35 g/L)	…	
REV-PVP (HU)		
≤ 20 HU	Reference	
> 20 HU	3.315 (1.917–5.733)	<0.001
RER-PVP		
≤ 0.716	Reference	…
> 0.716	…	
Necrosis		
Absent	Reference	
Present	1.938 (1.226–3.063)	0.005
Peripancreatic fat infiltration		
Absent	Reference	
Present	1.714 (1.055–2.783)	0.030

Data in parentheses are 95% CIs. Ellipsis indicates p value is not significant and should be excluded from the multivariate Cox model.

PFS, progression-free survival; CI, confidence interval; HR, hazard ratio; CA19-9, carbohydrate antigen 19-9; REV, relative enhanced value; RER, relative enhanced ratio; PVP, portal venous phase.

^*^Variables with p < 0.05 in univariate analysis were applied to multivariate analysis using a stepwise Cox proportional hazards regression mode.

**Figure 5:**
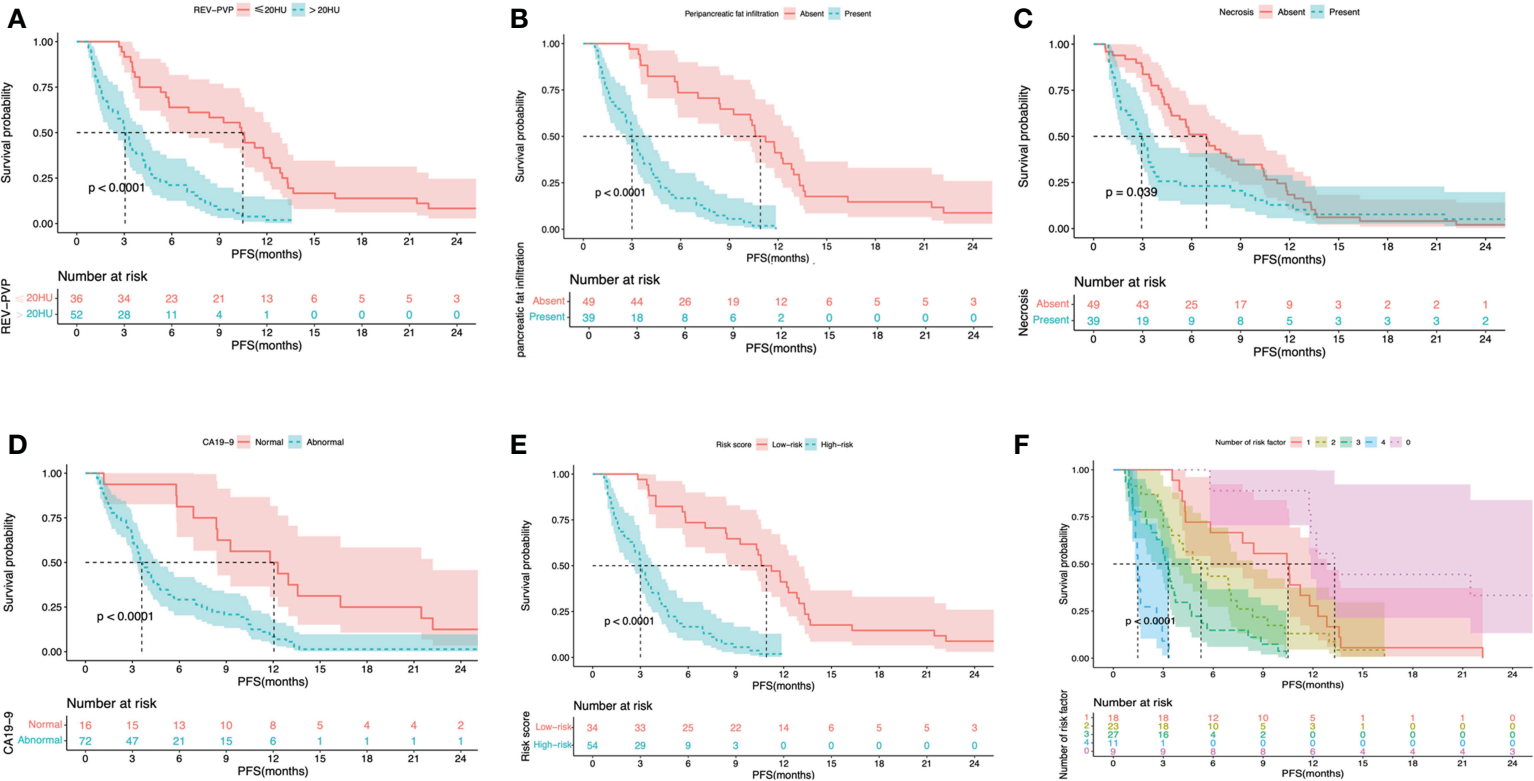
Kaplan-Meier survival curves shown PFS according to the REV-PVP (≤20 HU or >20 HU) **(A)**, peripancreatic fat infiltration (absent or present) **(B)**, necrosis (absent or present) **(C)**, serum CA19-9 level (normal or abnormal) **(D)**, nomogram-predicted high- or low-risk **(E)**, and the number of risk factor **(F)**. PFS, progression-free survival; REV, relative enhanced value; PVP, portal venous phase; CA19-9, carbohydrate antigen 19-9.

The results of Kaplan-Meier survival analysis based on the above four risk factors were shown in **Table 4** and **Figures 5A–D**. LAPC patients with REV-PVP > 20 HU progressed significantly faster than those with REV-PVP ≤ 20 HU (median PFS: 10.4 months *vs*. 3.0 months, *p* < 0.001). The median PFS of patients with peripancreatic fat infiltration, necrosis, and abnormal serum CA19-9 level was significantly shorter than those without peripancreatic fat infiltration (3.0 months *vs*. 5.8 months), necrosis (2.9 months *vs*. 6.9 months), and normal CA19-9 level (3.5 months *vs*. 11.8 months) (all *p <*0.05). The survival analysis of different chemotherapy regimens is shown in **Supplementary Table S1** and **Figure S1**.

**Table 4:** Kaplan-Meier analysis for PFS stratified by risk factors.

Variables (n, %) ^*^	Median PFS (months) (95% CI)	Log-Rank *p* Value
REV-PVP		
≤ 20 HU (n=21, 23.9)	10.4 (8.4–12.4)	<0.001
> 20 HU (n=67, 76.1)	3.0 (2.3–3.7)	
Peripancreatic fat infiltration		
Absent (n=49, 55.7)	5.8 (2.7–9.0)	0.001
Present (n=39, 44.3)	3.0 (2.0–4.0)	
Necrosis		
Absent (n=49, 55.7)	6.9 (4.9–8.0)	0.039
Present (n=39, 44.3)	2.9 (2.3–3.5)	
CA19-9 level		
Normal (n=16, 18.2)	11.8 (5.9–17.7)	<0.001
Abnormal (n=72, 81.8)	3.5 (2.9–4.2)	
Nomogram predicted risk		
Low-risk (n=34, 38.6)	10.6 (8.6–12.6)	<0.001
High-risk (n=54, 61.4)	3.0 (2.3–3.7)	

PFS, progression-free survival; CI, confidence interval; REV, relative enhanced value; RER, relative enhanced ratio; PVP, portal venous phase; CA19-9, carbohydrate antigen 19-9.

^*^The content in parentheses of parameter indicates the number and percentage of patients.

### Nomogram development and evaluation

**Figure 6:**
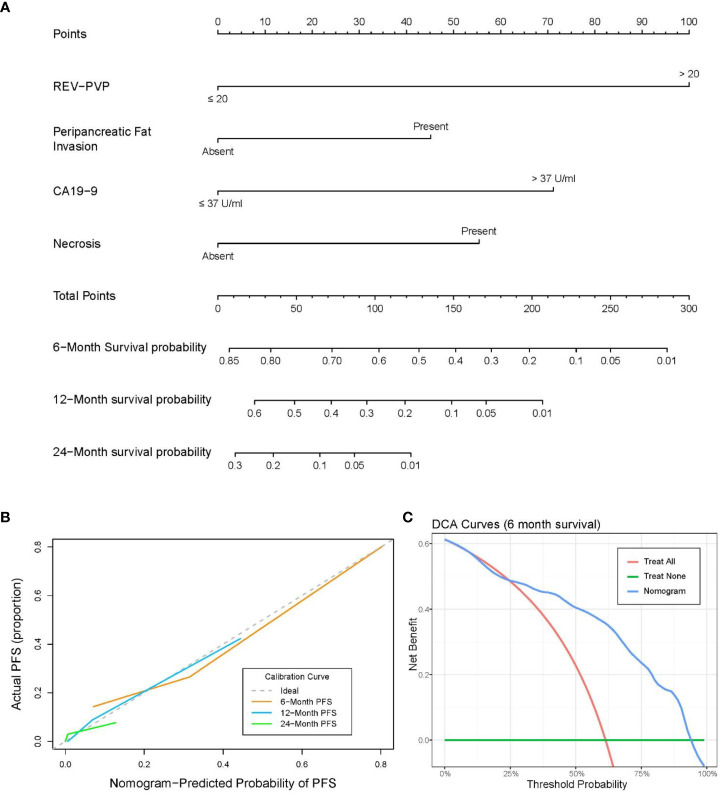
**(A)** Nomogram for predicting the 6-, 12-, and 24-month progression-free survival (PFS). **(B)** Calibration curve for PFS nomogram. **(C)** Decision-curve analysis (DCA) for the nomograms of 6-month PFS.

A nomogram integrating REV-PVP, peripancreatic fat infiltration, necrosis, and serum CA19-9 level, identified in multivariate Cox analysis, was constructed to predict 6-, 12-, and 24-month PFS for LAPC patients receiving IORT (**Figure 6A**). The prediction nomogram achieved a Harrell’s C-index of 0.779 (95% CI = 0.736–0.822), indicating an acceptable predictive capability for PFS. The calibration curves of the nomogram showed good agreement between the nomogram-predicted risk probabilities and the actual observed progression after IORT (**Figure 6B**). The clinical usefulness of the nomogram was evaluated *via* DCA, which indicated that when the threshold probability is between 25.0% and 93.4%, the prediction nomogram of 6-month will have a net benefit from IORT (**Figure 6C**).

### Progression risk stratification based on the nomogram

**Figure 7:**
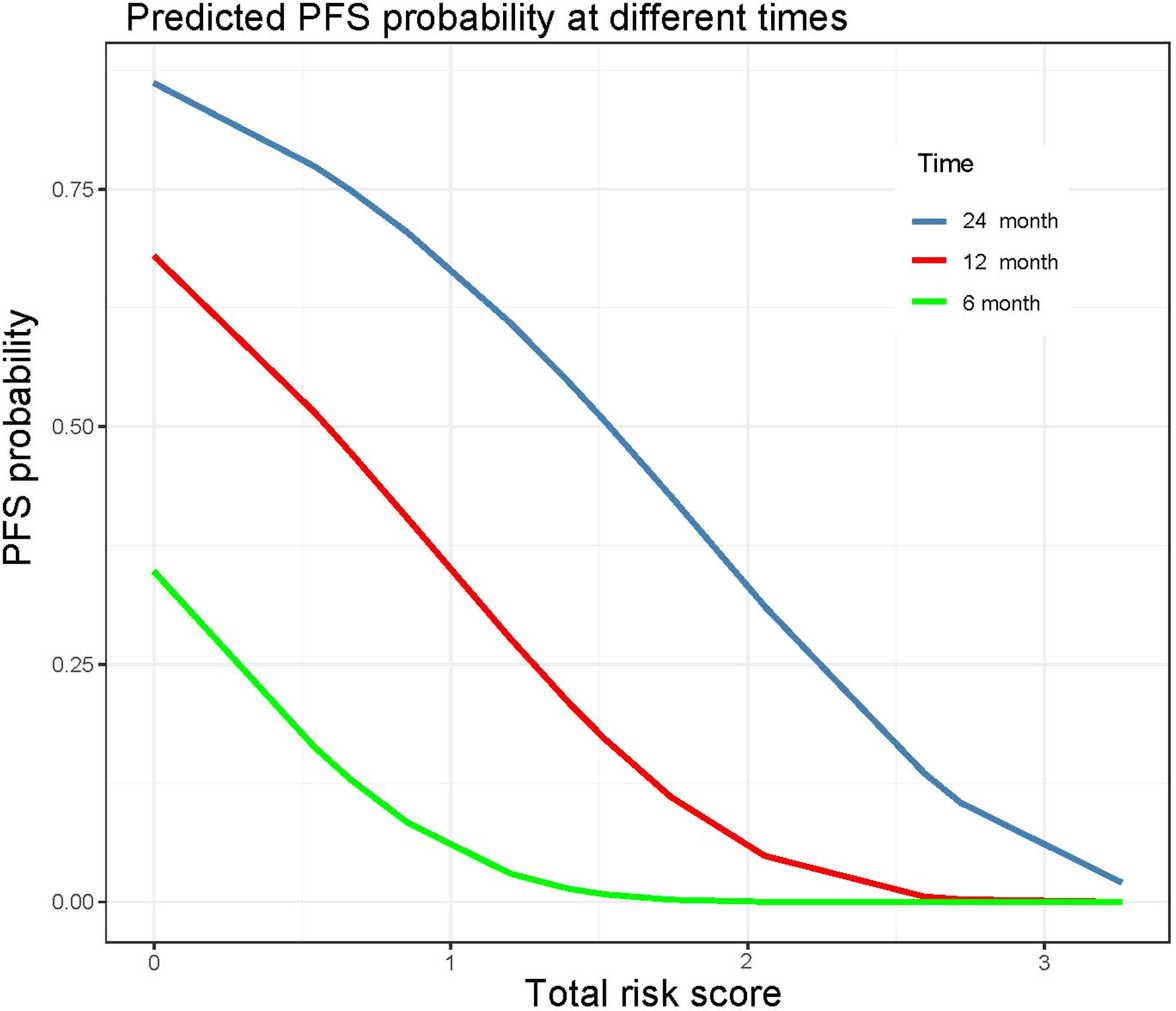
Probability of 6-, 12-, and 24-month progression-free survival according to the preoperative total risk score.

A risk-scoring system was constructed with the independent risk factors and their regression coefficients in multivariate Cox analysis for predicting the progression of LAPC receiving IORT. The formula was as follows: Risk score = REV-PVP (>20 HU) × 1.199 + peripancreatic fat infiltration (present)× 0.539 + CA19-9 (>37 U/mL) × 0.853 + necrosis (present) × 0.661. The risk score ranged from 0 to 3.252, and the relationship between risk score and predicted PFS probability is shown in **Figure 7**.

The optimal cut-off value of the risk-score was 1.52, which stratifies the risk of progression into two groups: low-risk group (risk-score > 1.52; 34/88, 38.6%) and high-risk group (risk-score ≤ 1.52; 54/88, 61.4%). LAPC patients with high-risk progressed significantly faster than those with low-risk (median PFS: 3.0 months, 95% CI: 2.3–3.7 months *vs*. 10.6 months, 95% CI = 8.6–12.6 months, *p* < 0.001) (**Table 4** and **Figure 5E**). The 3-month, 6-month, and 1-year PFS rates were 97.1%, 73.5%, and 41.2% in the low-risk group, while 53.7%, 16.7%, and 0% in the high-risk group, respectively.

### Predictive performance of risk factors and nomogram

Validation of any combination of the risk factors was performed and displayed in **Table 5** and **Figure 5F**. Further stratified comparisons revealed that the difference in PFS among each other was also statistically significant (**Supplementary Table S7**). The results showed that patients with all four risk factors progressed most rapidly and had the worst median PFS (1.5 months, 95% CI = 1.2–1.7 months), with PFS rates at 3-month, 6-month, and 1-year of 11.1%, 0.0%, and 0.0%, respectively. In comparison, patients with none of the risk factors showed the longest median PFS (13.3 months, 95% CI = 10.1–16.5 months), with PFS rates at 3-month, 6-month, and 1-year of 100.0%, 88.9%, and 66.7%, respectively.

**Table 5:** Correlation of number of independently predictive factors with progression-free survival.

Number of Risk Factors ^*^ (n, %)	Cox proportional hazards regression analysis		PFS rate at different time			Median PFS (months) (95% CI)
	HR (95% CI)	*p* Value	3-month (%)	6-month (%)	1-year (%)	
0 (n=9, 10)	Reference		100	88.9	66.6	13.3 (10.1–16.5)
1 (n=18, 20)	3.1(1. 2, 8.0)	0.02	100	66.7	27.7	10.4 (6.0–14.7)
2 (n=23, 23)	6.0 (2.3, 15.6)	< 0.001	78.3	43.5	13.0	5.3 (3.0–7.6)
3 (n=27, 13)	14.7 (5.4, 39.8)	< 0.001	59.3	14.8	0.0	3.3 (2.7–3.9)
4 (n=11, 13)	57.9 (17.6, 191.4)	< 0.001	11.1	0.0	0.0	1.5 (1.2–1.7)

PFS, progression-free survival; HR, hazard ratio; CI, confidence interval.

^*^Risk factors include REV-PVP (> 20HU), peripancreatic fat infiltration, abnormal CA19-9 level, and necrosis. The content in parentheses of parameter indicates the number and percentage of patients.

The predictive performance of PFS probabilities at different times calculated according to the nomogram is listed in **Table 6**. The accuracy of predictive PFS ranged from 64.8% to 79.5% at 6-months, from 78.4% to 87.5% at 1-years, and from 90.9% to 92.0% at 2-year, respectively. The nomogram exhibited the best performance when predicting PFS probability greater than 60% at 6-month, with the highest F1 score, accuracy, sensitivity, and specificity of 0.74, 79.5%, 73.5%, and 83.3%, respectively.

**Table 6:** Prediction performance for PFS of nomogram.

Time	PFS probability	Accuracy (%)	Sensitivity (%)	Specificity (%)	PPV (%)	NPV (%)	F1 Score
6 months	≥ 90%	64.8 (53.9–74.7) [57/88]	14.7 (5.0–31.1) [5/34]	96.3 (87.3–99.5) [52/54]	71.4 (29.0–96.3) [5/7]	66.7 (55.3–76.8) [54/81]	0.24
	≥ 60%	79.5 (69.6–87.4) [70/88]	73.5 (55.6–87.1) [25/34]	83.3 (70.7–92.0) [45/54]	73.5 (55.6–87.1) [25/34]	83.3 (70.7–92.0) [45/54]	0.74
	≥ 30%	71.5 (70.0–80.7) [63/88]	88.2 (73.3–95.3) [30/34]	61.1 (46.9–74.0) [33/54]	58.8 (44.2–72.4) [30/51]	89.2 (74.6–97.0) [33/37]	0.71
1 year	≥ 60%	84.1 (74.8–91.0) [74/88]	21.4 (4.7–50.8) [3/14]	88.6 (95.9–99.2) [71/74]	50.0 (11.8–88.2) [3/6]	86.6 (77.2–93.1) [71/82]	0.30
	≥ 40%	87.5 (78.7–93.6) [77/88]	50.0 (23.0– 77.0) [7/14]	94.6 (86.7–98.5) [70/74]	63.6 (30.8–89.1) [7/11]	90.9 (82.2–96.3) [70/77]	0.56
	≥ 20%	78.4 (68.4–86.5) [69/88]	85.7 (57.2–98.2) [12/14]	77.0 (65.8–86.0) [57/74]	41.4 (23.5–61.1) [12/29]	96.6 (88.5–99.1) [57/59]	0.56
2 years	≥ 30%	92.0 (84.3–96.7) [81/88]	33.3 (0.8–90.6) [1/3]	94.1 (86.8–98.0) [80/85]	16.7 (4.2– 64.1) [1/6]	97.6 (91.5–99.7) [80/82]	0.22
	≥ 15%	90.9 (82.9–96.0) [80/88]	33.3 (0.8–90.6) [1/3]	92.9 (85.2–97.3) [79/85]	14.3(3.6–57.9) [1/7]	97.5 (91.4–99.7) [79/81]	0.20

Data are percentages with 95% CIs in parentheses and numbers of observations in brackets.

PFS, progression-free survival; CI, confidence interval; PPV, positive predictive value; NPV, negative predictive value.

## Discussion

In this study, we discovered that the baseline REV-PVP, peripancreatic fat infiltration, necrosis, and serum CA19-9 were independent risk factors for progression in LAPC patients after IORT. A risk prediction nomogram was constructed based on the above CT imaging features and CA19-9, with an excellent predictive performance for PFS (C-index of 0.779). This provides a potential noninvasive and simple approach to assist clinicians in identifying candidates who might benefit from IORT before treatment and achieving an individualized treatment.

The PFS time for the whole cohort in this study was 4.3 months, which was a little shorter than previous studies reported in a meta-analysis (4). The possible explanations might be due to no radical surgical resection performed after IORT, relatively late tumor stage and poor physical conditions of the patients. In a review of chemotherapy and radiotherapy for LAPC without surgery (31), the PFS times ranged from 2.1 to 7.6 months, which were partially in line with our result. Ogawa K et al. found IORT combined with chemotherapy obtained a survival benefit compared with that of IORT alone (9). Furthermore, IORT could improve local control rate and relieve pain substantially, so it is recommended to be performed in patients with LAPC (10).

Our results revealed that the simple CT quantitative parameter REV-PVP could be used as an objective imaging marker for progression prediction in LAPC after IORT, which was calculated based on the relative enhancement values between the primary tumor and pancreatic parenchyma. LAPC patients with high REV-PVP (>20HU), meaning a lower tumor attenuation compared with adjacent pancreatic parenchyma, progressed significantly more quickly than patients with lower REV-PVP (≤20HU). High REV-PVP implied the CT attenuation difference between pancreatic parenchyma and tumor was great, in other words, a relatively low CT attenuation of tumor itself. Previous studies found that a hypo-attenuated tumor on the CECT indicated poor differentiation of PDAC, in which cancer cells proliferated rapidly and probably lead to the insufficient blood supply and consequently more areas of necrosis (32, 33). In contrast, iso-attenuated lesions or lesions with enhancement closer to surrounding pancreatic parenchyma are probably well- or moderately-differentiated, with more residual alveolar cells and closer to normal pancreatic tissue (33). Furthermore, PDAC appearing as hypo-attenuated may be associated with an extensive desmoplastic stromal reaction, resulting in decreased blood flow and insufficiency of blood supply (34). Moreover, dense fibrotic deposition also causes hypoxia, which is an important cause of resistance to radiotherapy (35, 36). Therefore, we speculated that low CT attenuation of tumor or high REV-PVP might indicate a more aggressive LAPC, less sensitivity to IORT, and a poorer prognosis. Shin et al. proposed PDAC with longer overall survival (OS) was associated with hyper-attenuation in resectable/borderline resectable/locally advanced pancreatic cancer (37), which is in line with our results. Our study just focused on patients with LAPC receiving IORT and utilized relative enhancement CT values between the tumor and the surrounding parenchyma instead of absolute values, avoiding the influence of hemodynamics and individual differences. Cai et al. also reported that high-delta-3 (differences in tumor and surrounding parenchymal attenuation coefficients at pancreatic phase) PDACs corresponded more often with aggressive histologic grade, larger tumor size, less extensive fibrous stromal fraction, and poor disease-free survival and OS (16). The advantage of our study was that we directly measured quantitative CT values at the maximum cross-section, which was more practical for clinical use.

Additionally, our research also supported the evidence that peripancreatic fat infiltration and necrosis, two semantic features of imaging, were indicators for poor prognosis, in accordance with previous studies (15, 38). Peripancreatic fat infiltration might reflect the extent of tumor invasion, not simple a desmoplastic reaction or edema (13, 15, 39). The presence of peripancreatic fat infiltration reduces the chance of R0 resection and leads to poor survival outcomes (13, 40). Necrosis correlates with a higher degree of malignancy in the tumor, which implies rapid proliferation of the tumor cells, leading to tissue ischemia and hypoxia (38). As known to all, hypoxia was a major cause of radiotherapy resistance (35, 36). Therefore, the presence of necrosis is considered as a poor prognostic imaging indicator in LAPC.

CA19-9 is the most important serological biomarker in PADC, which is also reported to be correlated with tumor burden and prognosis (15). In this study, we investigated the value of CA19-9 in prognosis prediction and demonstrated that high baseline serum CA19-9 levels could be used as an indicator of a short PFS time. So, we added CA19-9 to the nomogram to improve the prediction performance.

We constructed a combined nomogram that incorporates CT imaging features and CA19-9 for progression prediction in LAPC at the individual level. The results indicated that the nomogram showed satisfactory predictive accuracy, with a C-index of 0.779. Using this nomogram to predict no progression probability over 60% at 6-month, the F1 score, accuracy, sensitivity, and specificity could be achieved at 0.74, 79.5%, 73.5%, and 83.3%, respectively. This nomogram might show great clinical utility in predicting progression and identifying optimal candidates in LAPC prior to IORT using a simple and practical method. LAPC patients with a low risk of progression would be suitable for and benefit from IORT, whereas in the high-risk group, radiotherapy might be less effective and other treatment strategies might be considered to improve the patient’s prognosis.

We should note that our study has several limitations. First, in order to accurately find the most suitable patients for IORT, we only used the progression as the endpoint and did not include OS. The prediction ability of the nomogram for OS in LPAC patients receiving IORT needs to be further clarified in our future work. Second, the patients received different treatment modalities after IORT, which to some extent, may have introduced some bias. But it is consistent with the clinical fact that the treatment of LAPC is highly individual. Third, the recruited patients were from a single institution, and the sample size was small, so no validation of the nomogram was performed. A larger sample size from multicenter is needed to further validate our results. Fourth, the Lewis antigen status was not considered in this study. The nomogram could not be applied directly to Lewis antigen negative individuals. Further validation in an independent cohort of Lewis negative patients was needed. Finally, the CT scanners in this study were diverse. However, it might broaden the scope of application and compensate for the insufficiency of the single-center study to some extent.

## Conclusion

In conclusion, CT imaging features and serum CA19-9 provide a tool for predicting progression in patients with LAPC receiving IORT. We constructed and proposed a simple and practical combined nomogram to stratify the risk of progression and identify suitable candidates for IORT before treatment in LAPC. Moreover, the nomogram that integrates baseline CT features and serum CA19-9 might serve as an effective tool in routine clinical practice to help clinicians identify patients who might benefit from IORT and make proper treatment decisions preoperatively and individually. A multicenter prospective study will be needed to further validate the p7otential predictive value of the nomogram in the future.

## Data availability statement

The raw data supporting the conclusions of this article will be made available by the authors, without undue reservation.

## Ethics statement

This retrospective study was approved by the Institutional Review Board of National Cancer Center/Cancer Hospital, Chinese Academy of Medical Sciences (No. 21/412-2608). The ethics committee waived the requirement of written informed consent for participation.

## Author contributions

Conceptualization, WC, YZ, DL, XZ, and XM; Data curation, WC, YZ, ZT, DL, QF, ZJ, RC, and ZC; Formal analysis, WC, YZ, ZT, DL, XZ, and XM; Methodology, WC, YZ, QF, ZJ, RC, ZC, and SL; Software, WC, YZ, RC, ZC, and SL; Validation, ZT, DL, QF, ZJ, and SL; Writing—original draft, all authors; Writing—review and editing, all authors; Supervision, XZ and XM. All authors contributed to the article and approved the submitted version.
