# Supplementary material for: Combined CT and serum CA19-9 for stratifying risk for progression in patients with locally advanced pancreatic cancer receiving intraoperative radiotherapy
Source: Front Oncol. 2023 Apr 14;13:1155555. doi: 10.3389/fonc.2023.1155555 (PMC10140514; doi:10.3389/fonc.2023.1155555)
Supplement: Supplementary file 1 [file DataSheet_1.pdf]

Supplementary Material

1. Materials and methods

1.1 Appendix S1: IORT and adjuvant therapy

A multidisciplinary team, which included surgeons, radiation oncologists, and radiologists, determined the appropriate treatment strategy of IORT and the following adjuvant therapy in LAPC patients. During surgery, a cytological diagnosis and a gross examination were routinely performed to confirm the diagnosis of pancreatic cancer without peritoneal or liver metastases. Before IORT, palliative surgery, including gastrojejunostomy or biliary bypass as appropriate, was regularly carried out depending on the tumor's location and clinical symptoms. After palliative surgery, the extent of lesion at operation was accessed by the surgeon and radiation oncologist, then a field including the pancreatic mass with a 0.5-1.0cm margin around and regional lymph nodes was covered by a cone[7]. The electron energy of electron beam employed in IORT was 9-12 Mev depending on the maximum diameter of tumor, with an average dose of 14.8 Gy (range 13.5-15.0 Gy). In order to protect adjacent normal tissue, the IORT field excluded the liver, gastric wall and small bowels.

Sequential adjuvant therapy was composed of chemoradiation or chemotherapy, depending a multidisciplinary team discussion. Postoperative Radiotherapy adopt external beam radiation therapy, which the regimen was 1.8–2.5Gy/fraction and total dose of 45–54Gy. Due to the high toxicity of FOLFIRINOX regimens, chemotherapy in this study adopted gemcitabine-based regimens (Gemcitabine, Gemcitabine plus albumin-bound paclitaxel, or Gemcitabine plus S-1).

1.2 Appendix S2: Image analysis

For the measurement of CT quantitative parameters, two regions of interest (ROI) were manually placed to measure the quantitative parameters as the following principles: one freehand ROI contained as much as lesion in the section of the largest tumor diameter avoiding adjacent vasculature and calcifications, another round ROI on the surrounding normal pancreatic parenchyma on the same or adjacent slice. An example and illustration of measurement of REV and RER were shown in **Figure 2**. The results of all quantitative parameters were recorded as the average of the two measurements.

The CT quantitative parameters were calculated using the following equations, CT_{tumor} indicates the CT attenuation value of tumor, $CT_{pancreas}$ indicates the CT attenuation value of surrounding normal peripancreatic parenchyma:

$$REV = CT_{pancreas} - CT_{tumor}$$

$$RER = \frac{CT_{tumor}}{CT_{pancreas}}$$

2. Supplementary tables

2.1 Table S1. Median PFS Stratified by chemotherapy regimen

**Chemotherapy regimen**	**N (%)** * (Total, N=56)	**Univariate analysis**		**Median PFS (months) (95% CI)**
		**HR (95% CI)**	***p* Value**	
Gemcitabine	13 (23.2)	Reference	0.011	2.3 (0.0 - 4.8)
Gemcitabine plus Albumin-bound paclitaxel	28 (50.0)	2.348 (1.270–4.341)		4.7 (1.3 -8.3)
Gemcitabine plus S-1	15 (26.8)	0.444(0.204–0.967)	0.041	4.3 (3.3 - 5.3)

Abbreviations: *PFS*, progression-free survival; *CI*, confidence interval.

* Data are expressed as the number of patients and percentage in parentheses.

2.2 Table S2. Multiphase contrast-enhanced CT scan parameters

Parameters	Revolution CT	Discovery CT 750 HD	Lightspeed VCT	Optima CT 660
No. of channels	256	64	64	64
Section collimation	128 × 0.625	64 × 0.625	64 × 0.625	64 × 0.625
Thickness (mm)	5	5	5	5
Interval (nm)	5	5	5	5
Helical pitch	0.992	0.984	0.984	1.375
Gantry rotation time(s)	0.5	0.7	0.5	0.5
Tube voltage (KVP)	120	120	120	120
Matrix	512 × 512	512 × 512	512 × 512	512 × 512

2.3 Table S3. Definition of each CT imaging feature in this study

CT imaging features	Definitions	Example of imaging features
Necrosis	Tumoral tissue that did not enhance on multiphasic CT examinations after contrast medium injection	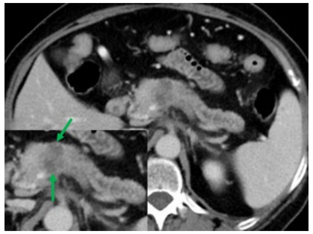
Rim-enhancement	irregular ringlike enhancement with a relatively hypo-vascular central area on dynamic-enhanced images	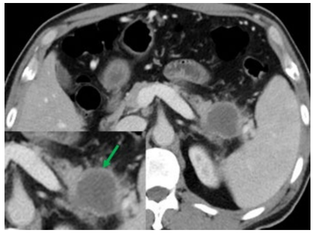
Peripancreatic fat infiltration	Tumor directly attached to the peri-pancreatic adipose tissues or extending directly from the intra-pancreatic tumor to the extra-pancreatic adipose tissues resulting in blurring or hyperdense shadowing at the interface between the tumor and the adipose tissues	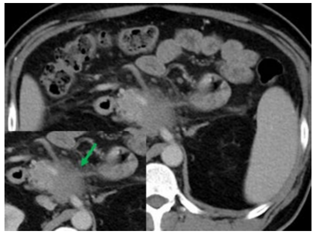

Suspicious lymph nodes	Including any of the following: short axis > 1 cm, abnormal round morphology, heterogeneity, or central necrosis.	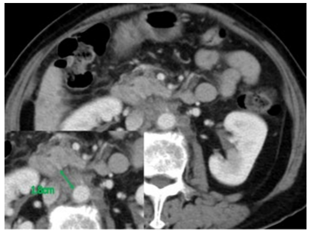
Pancreatic duct dilatation	Interrupted main pancreatic duct was defined as an abrupt luminal disruption of the main pancreatic duct.	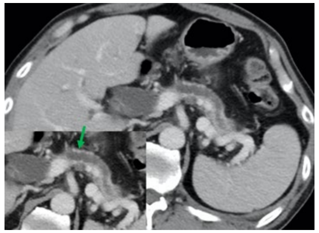
Atrophic upstream pancreatic parenchyma	A main pancreatic duct calibre to total pancreatic parenchymal width ratio of less than 0.50.	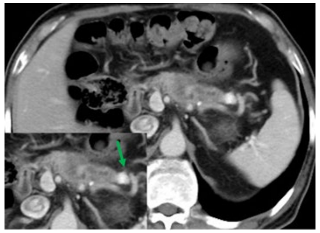

The green arrows indicated the typical sign of the imaging features.

References:[1-4]

1.Lee S, Kim SH, Park HK, et al. Pancreatic Ductal Adenocarcinoma: Rim Enhancement at MR Imaging Predicts Prognosis after Curative Resection. *Radiology* 2018;288(2):456-66. doi: 10.1148/radiol.2018172331.

2.Tamada T, Ito K, Kanomata N, et al. Pancreatic adenocarcinomas without secondary signs on multiphasic multidetector CT: association with clinical and histopathologic features. *Eur Radiol* 2016;26(3):646-55. doi: 10.1007/s00330-015-3880-3.

3.AlHawary MM FI, Chari ST, Fishman EK, Hough DM, Lu DS, Macari M, Megibow AJ, Miller FH, Mortele KJ, Merchant NB, Minter RM, Tamm EP, Sahani DV,

Simeone DM. . Pancreatic ductal adenocarcinoma radiology reporting template: consensus statement of the society of abdominal radiology and the american pancreatic association. *Gastroenterology* 2014;146(1):291-304. doi: 10.1053/j.gastro.2013.11.0042.
4. Yoon SH LJ, Cho JY, Lee KB, Kim JE, Moon SK, Kim SJ, Baek JH, Kim SH, Kim SH, Lee JY, Han JK, Choi BI. Small (≤ 20 mm) pancreatic adenocarcinomas: analysis of enhancement patterns and secondary signs with multiphasic multidetector CT. *Radiology* 2011;259:442–52. doi: 10.1148/radiol.11101133

2.4 Table S4. **Median PFS Stratified by Progression pattern**

Progression pattern	N (%)	Median PFS (months) (95% CI)
Distant metastasis	52/88 (59.1%)	4.3 (2.4-6.2)
Liver	27	3.4 (2.5-4.2)
Lung	16	5.8 (0.3-11.5)
Others [*]	9	4.3 (2.4-6.1)
Local progression	19/88 (21.6%)	7.1(0.5-13.7)
Both [*]	17/88 (19.3%)	3.5(2.1-4.9)

Abbreviations: *PFS*, progression-free survival; *CI*, confidence interval.

[*] Others include peritoneal, bone, brain, adrenal gland, or non-regional lymph node metastasis.

[**] Both indicate distant metastasis and local progression.

2.5 Table S5. **The relationship between tumors and peripheral vascular**

**Tumor location**	**Head/uncinate N (%)**	**Body/tail N (%)**
Total number	63 (71.6)	25 (28.4)
Solid tumor contact >180° with the SMA	35 (39.8)	13 (14.8)
Solid tumor contact >180° with the CA	21 (23.9)	7 (7.9)
Solid tumor contacts with the CA and aortic involvement		3 (3.4)
Unreconstructible SMV/PV due to tumor involvement or occlusion (can be due to tumor or bland thrombus).	7 (7.9)	2 (2.3)

Data are expressed as the number of patients and percentage in parentheses.

Abbreviations: *CA*, celiac axis; *SMA*, superior mesenteric artery; *CHA*, or common hepatic artery.

2.6 Table S6. Interobserver agreement of CT Semantic features and quantitative parameters

Variables	kappa coefficient or ICC (95% CI)
**CT quantitative parameters**	
long-axis(cm)	0.87 (0.82–0.91)
short-axis(cm)	0.84 (0.80–0.92)
REV-AP(HU)	0.85 (0.80–0.91)
REV-PPP(HU)	0.84 (0.80–0.89)
REV-PVP(HU)	0.86 (0.81–0.89)
RER-AP	0.85 (0.80–0.91)
RER-PPP	0.84 (0.80–0.89)
RER-PVP	0.86 (0.81–0.89)
**CT Semantic features**	
Tumor location	1.00 (1.00–1.00)
Tumor attenuating in N	0.75 (0.69–0.86)
Tumor attenuating in AP	0.70 (0.61–0.85)
Tumor attenuating in PPP	0.74 (0.65–0.83)
Tumor attenuating in PVP	0.71 (0.64–0.81)
Necrosis	0.82 (0.79–0.89)
Rim-enhancement	0.69 (0.62–0.76)
Peripancreatic fat infiltration	0.74 (0.63–0.86)
Suspicious lymph nodes	0.65 (0.60–0.69)
Pancreatic duct dilatation	0.70 (0.63–0.75)
Atrophic up stream pancreatic parenchyma	0.72 (0.67–0.78)

Data are Cohens κ values and ICC values; numbers in parentheses are 95% CI.

Abbreviations: *ICC*, intraclass correlation coefficient; *CI*, confidence interval; *REV*, relative enhanced value; *RER*, relative enhanced ratio; *N*, non-enhanced; *AP*, arterial phase; *PPP*, pancreatic parenchymal phase; *PVP*, portal venous phase.

2.7 Table S7. Pairwise Comparisons in groups of Number of Independent Predictive Factors

**Number of Factors**	Log-rank test	0	1	2	3	4
0	Chi-square*		6.5	12.2	22.7	20.4
	p Value		0.011	0.000	0.000	0.000
1	Chi-square*	6.5		4.1	22.4	37.7
	p Value	0.011		0.042	0.000	0.000
2	Chi-square*	12.2	4.1		5.3	22.7
	p Value	0.000	0.042		0.021	0.000
3	Chi-square*	22.7	22.4	5.3		9.9
	p Value	0.000	0.000	0.021		0.002
4	Chi-square*	20.4	37.7	22.7	9.9	
	p Value	0.000	0.000	0.000	0.002	

Risk factors include REV-PVP (≥ 20HU), peripancreatic fat infiltration, necrosis, and abnormal CA19-9 level.

* Chi-square is the statistic of log-rank test.

3. Supplementary Figures

3.1 Figure S1

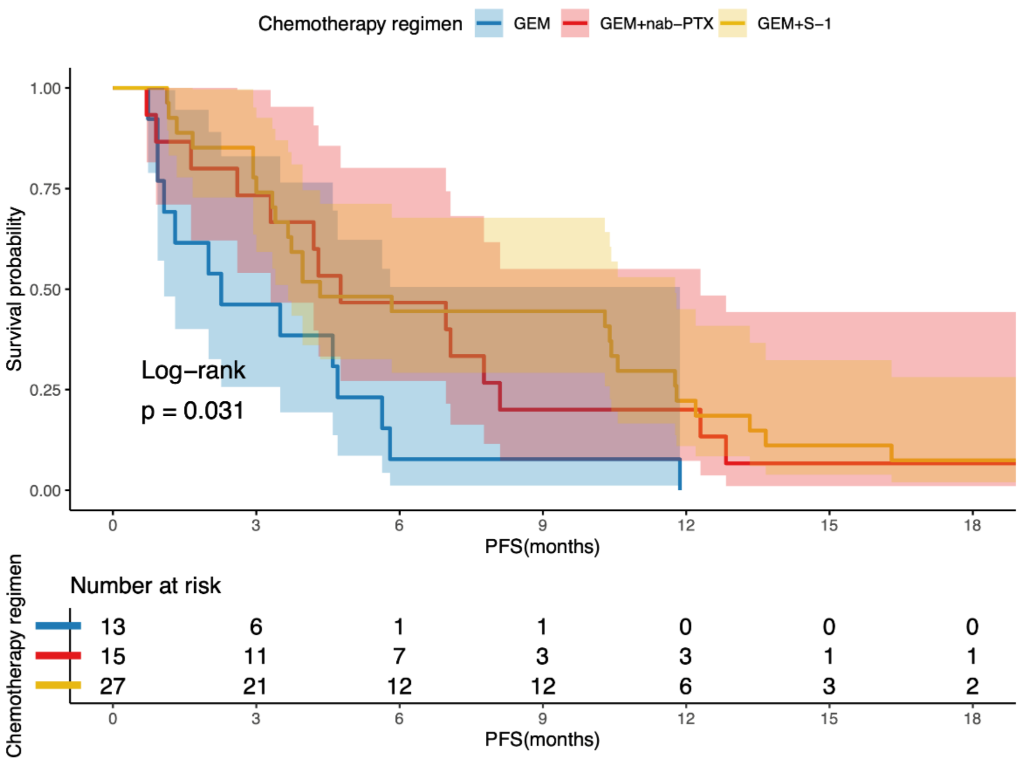

Figure S1. Kaplan-Meier survival curves shown PFS according to the chemotherapy regimen. Abbreviations: *PFS*, progression-free survival; *GEM*, Gemcitabine; *GEM+nab-PTX*, Gemcitabine plus Albumin-bound paclitaxel; *GEM+S-1*, Gemcitabine plus S-1.
